# TGF-beta inhibitors in cancer therapy: a review of the TGF-beta signal transduction pathway and current developments

**DOI:** 10.17179/excli2026-9381

**Published:** 2026-04-28

**Authors:** Shun-Ban Tai, Jenq-Lin Yang, Pei-Feng Liu, Chun-Lin Chen

**Affiliations:** 1 Division of Rheumatology, Immunology and Allergy, Department of Internal Medicine, Zuoying Armed Forces General Hospital, Kaohsiung 81342, Taiwan ROC; 2 Institute for Translational Research in Biomedicine, Kaohsiung Chang Gung Memorial Hospital, Kaohsiung 83301, Taiwan ROC; 3 Department of Biomedical Science and Environmental Biology, Kaohsiung Medical University, Kaohsiung 80756, Taiwan ROC; 4 Department of Biological Sciences, National Sun Yat-Sen University, Kaohsiung 80424, Taiwan ROC; 5 National Museum of Marine Biology and Aquarium, Pingtung 94450, Taiwan ROC; 6 Graduate Institute of Natural Products, Kaohsiung Medical University, Kaohsiung 80756, Taiwan ROC; 7 Department of Biotechnology, Kaohsiung Medical University, Kaohsiung 80756, Taiwan ROC

**Keywords:** TGF-beta signaling, cancer therapy, TGF-beta inhibitors, tumor microenvironment, drug repurposing, combination therapy

## Abstract

Transforming growth factor-β (TGF-β) belongs to a family of structurally and functionally related cytokines that play essential roles in embryonic development, tissue homeostasis, and cell fate regulation. Dysregulation of TGF-β signaling contributes to a broad spectrum of diseases, including cancer, fibrosis, and immune disorders. In cancer, TGF-β exhibits a context-dependent dual role, functioning as a tumor suppressor during early stages while promoting invasion, metastasis, escape from immune surveillance, and tumor microenvironment remodeling in advanced-stage cancer through effects on stromal cells, extracellular matrix deposition, and angiogenesis. This functional duality makes therapeutic targeting both attractive and challenging. Although current strategies mainly focus on ligand neutralization or receptor kinase inhibition, accumulating evidence indicates that TGF-β activity is also regulated beyond the receptor level, including receptor trafficking, co-receptor function, nucleocytoplasmic shuttling, indirect pathway modulation, and epigenetic regulation. In this review, we emphasize regulatory mechanisms that can be modulated by existing drugs, clinical candidates, or experimentally tractable compounds, rather than providing an exhaustive overview of the broader regulatory landscape of TGF-β signaling. We further highlight opportunities for drug repurposing and discuss how synergistic combination strategies may improve therapeutic efficacy and overcome resistance in TGF-β-driven cancers, supporting a broader therapeutic framework beyond canonical receptor inhibition.

See also the graphical abstract(Fig. 1).

## Introduction

**Figure 1:**
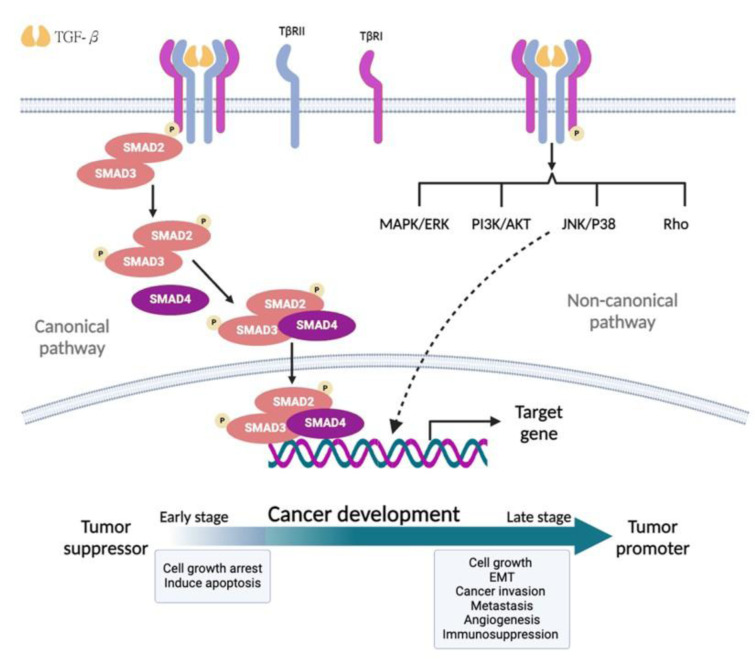
Graphical abstract TGF-β pathways and their role in tumorigenesis. TGF-β signaling could transduce through canonical or non-canonical pathways. Two types of TGF-β receptors, TGF-β type I (TβRI) and type II (TβRII) receptor, are responsible for ligand binding and signaling initiation. TGF-β ligands first recognize and bind to TβRII, which trigger receptor autophosphorylation and recruit TβRI to form a heteroreceptor complex. SMAD2/3 are phosphorylated by the kinase domain of activated TβRI, followed by interaction with SMAD4 and then be translocated into the nucleus to regulate expression of target genes. Apart from TGF-β/SMAD or canonical pathway, other pathways such as MAPK/ERK, PI3K/AKT, JNK/p38, and Rho pathway could also be induced by TGF-β stimulation. TGF-β pathway plays double-edged roles in cancer. As cancer develops, TGF-β switches its role from tumor suppressor to promoter, with loss of antiproliferative and proapoptotic properties and gains of abilities which could facilitate tumor growth and invasion.

Transforming growth factor-β (TGF-β) is a pleiotropic cytokine that orchestrates a wide range of physiological processes-from embryonic development to adult tissue homeostasis-by rewiring core cellular programs such as proliferation, differentiation, and apoptosis. Mammals express three TGF-β isoforms (TGF-β_1_, TGF-β_2_, and TGF-β_3_), which signal through a shared receptor system composed primarily of the TGF-β type II receptor (TβRII) and the type I receptor (TβRI/ALK5) (Tzavlaki and Moustakas, 2020[176]). In the canonical cascade, activation of mature TGF-β (after release from latency) allows high-affinity binding to TβRII, which recruits TβRI to form the signaling complex. TβRII then phosphorylates TβRI via its serine/threonine kinase domain, enabling TβRI to phosphorylate receptor-regulated SMADs (R-SMAD2/3). Phosphorylated R-SMADs complex with the common mediator SMAD4 and translocate to the nucleus to regulate target-gene expression (Tzavlaki and Moustakas, 2020[176]). In parallel with this SMAD pathway, the same receptor complex can engage non-canonical effectors-including AKT, ERK1/2, and p38/MAPK-which cooperate with SMAD signaling to shape cellular responses (Liu et al., 2018[111]) (see Figure 1(Fig. 1) (graphical abstract)). Dysregulation of these canonical and non-canonical branches contributes to tissue fibrosis, immune dysfunction, and oncogenesis (Leask and Abraham, 2004[98]; Prud'Homme, 2007[146]). Beyond the core TβRII-TβRI module, auxiliary receptors fine-tune ligand presentation, signal strength, and context specificity. The type III co-receptors betaglycan (TβRIII) and endoglin (CD105) modulate ligand capture and delivery to TβRII (Schoonderwoerd et al., 2020[155]; Duesman et al., 2023[50]). Importantly, a type V TGF-β receptor (TβR-V) has been described as a high-molecular-weight receptor mediating growth-inhibitory responses to TGF-β and IGFBP-3 in epithelial cells; in several contexts, TβR-V corresponds to LRP1 (low-density lipoprotein receptor-related protein-1) (Chen et al., 2021[24]; Baxter, 2023[8]; Yamamoto et al., 2024[186]). In addition, the cation-independent mannose-6-phosphate receptor (CI-M6PR) contributes to latent TGF-β activation and presentation at the cell surface, further shaping pathway availability and tone (Nykjaer et al., 1998[129]). Together, these auxiliary components create a modular architecture that explains the strong context dependence of TGF-β biology across tissues. Functionally, TGF-β acts as a double-edged sword in cancer-tumor-suppressive early, but pro-metastatic and immunosuppressive later (Akhurst and Derynck, 2001[1]). This has motivated therapeutic strategies ranging from ligand traps and antibodies to receptor-kinase inhibitors, trafficking-targeted modulators, and downstream pathway inhibitors, aiming to selectively dampen pro-tumor TGF-β programs while preserving essential homeostatic functions (Huang and Chen, 2012[78]; Wu et al., 2020[183]; Liu et al., 2021[112]; Teicher, 2021[172]). In this review, we structure the discussion with our laboratory's previous work as a conceptual backbone and extend the narrative by integrating complementary findings from other research groups, thereby providing a mechanism-focused and context-aware synthesis of TGF-β inhibition. In addition, the perspectives presented here are shaped by our current research interests and ongoing directions, particularly those aimed at understanding non-canonical regulatory layers of TGF-β signaling and their translational implications. By combining established knowledge with emerging mechanistic insights, this review seeks to highlight underexplored regulatory nodes that may expand future therapeutic strategies targeting TGF-β signaling. 

## Overview of TGF-β Signaling in Cancer

TGF-β primarily functions as a tumor suppressor by restraining cell proliferation, promoting apoptosis, and maintaining genomic stability. These cytostatic effects are mediated in part through the induction of cyclin-dependent kinase inhibitors such as p15, p21, and p57 (Seoane et al., 2001[158], 2002[157]; Chen et al., 2006[31]), as well as repression of growth-promoting genes including CDC25A, E2F1, and c-MYC (Pietenpol et al., 1990[143]; Schwarz et al., 1995[156]; Bhowmick et al., 2003[9]), collectively leading to cell cycle arrest and anti-mitogenic responses. TGF-β can also dampen early inflammatory signals and limit immune activation (Heath et al., 2000[71]), contributing to tissue homeostasis during early stages of tumorigenesis. During malignant progression, however, tumor cells frequently acquire resistance to the cytostatic and proapoptotic effects of TGF-β, often as a result of mutations or deletions in key pathway components such as TβRII, SMAD2, or SMAD4 (Markowitz et al., 1995[117]; Riggins et al., 1997[148]; Korkut et al., 2018[92]). Once these growth-inhibitory constraints are bypassed, tumor cells can exploit TGF-β signaling to promote epithelial-mesenchymal transition (EMT), invasion, and metastatic dissemination. Through both SMAD-dependent and non-SMAD pathways, TGF-β induces transcription factors including SNAIL, SLUG, TWIST, and ZEB family proteins, which drive loss of epithelial characteristics and acquisition of migratory phenotypes (Lamouille et al., 2014[97]). In parallel, TGF-β stimulates the expression of factors such as IL-11, PTHrP, and matrix metalloproteinases that facilitate extracellular matrix remodeling, tissue invasion, and organ-specific metastasis (Yin et al., 1999[192]; Wiercinska et al., 2011[182]; Pollari et al., 2012[145]), while angiogenic mediators including CTGF and VEGF contribute to neovascularization (Goumans et al., 2009[61]).

**Figure 2:**
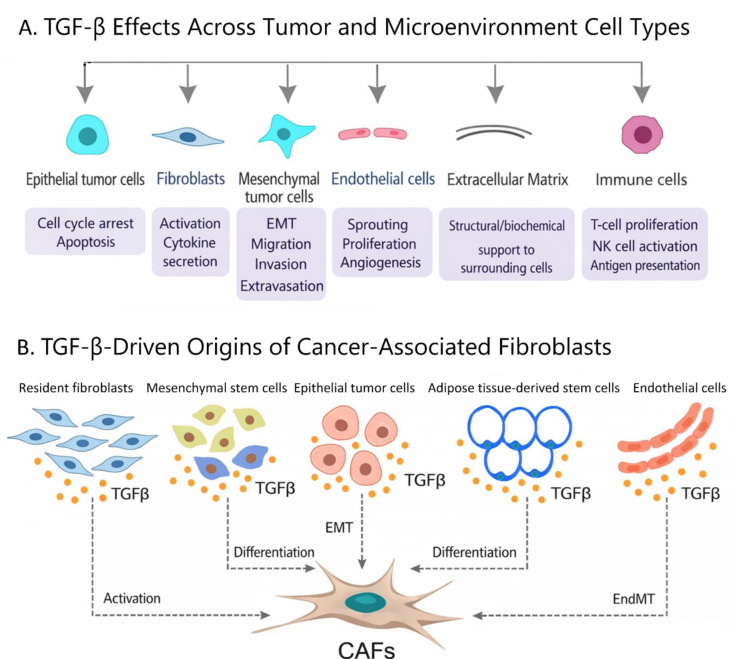
TGF-β Actions in the Tumor Microenvironment and CAF Induction Schematic summary of TGF-β functions across major tumor and stromal compartments. Panel A: Cell type-specific outcomes of TGF-β signaling in epithelial tumor cells (cell-cycle arrest/apoptosis in early contexts), fibroblasts (activation and cytokine secretion), mesenchymal tumor cells (EMT-associated migration/invasion/extravasation), endothelial cells (sprouting/proliferation/angiogenesis), extracellular matrix (structural and biochemical support), and immune cells (modulation of T-cell proliferation, NK-cell activation, and antigen presentation). Panel B: Major cellular sources contributing to cancer-associated fibroblast (CAF) generation under TGF-β-rich conditions. TGF-β activates resident fibroblasts and promotes differentiation of mesenchymal stem cells and adipose-derived stem cells into CAFs, while epithelial tumor cells and endothelial cells can contribute through EMT and EndMT, respectively.

Beyond its direct effects on tumor cells, TGF-β exerts profound influences on the tumor microenvironment (TME). The TME is composed of extracellular matrix, soluble factors, and diverse stromal and immune cell populations, and reciprocal interactions between tumor cells and stromal components critically shape disease progression (Liu et al., 2021[112]) (Figure 2A(Fig. 2)). Acting in a paracrine manner, TGF-β activates cancer-associated fibroblasts (CAFs), promotes extracellular matrix deposition, and enhances angiogenesis, thereby creating a microenvironment that supports tumor growth and limits drug penetration. TGF-β signaling is a major driver of CAF formation, promoting the activation of resident fibroblasts as well as the differentiation of mesenchymal stem cells, epithelial cells, and endothelial cells into fibroblast-like phenotypes (Evans et al., 2003[53]; Calon et al., 2014[16]) (Figure 2B(Fig. 2)). Dense CAF populations and the stiff extracellular matrix they generate can form physical barriers that reduce vascular perfusion and hinder the delivery of anticancer therapeutics, and excessive TGF-β activity within CAFs has been linked to resistance to immunotherapy.

TGF-β is a dominant regulator of immune function within the tumor microenvironment and contributes to the progressive suppression of antitumor immunity during tumorigenesis. As tumors develop, diverse myeloid populations-including myeloid-derived suppressor cells, macrophages, and neutrophils-accumulate and help establish an immunosuppressive milieu that limits effective T-cell responses (Ginefra et al., 2020[59]). Although dendritic cells normally initiate antitumor immunity by presenting tumor antigens to cytotoxic T lymphocytes and natural killer (NK) cells, TGF-β interferes with this process by impairing antigen presentation and inhibiting the activation and cytotoxic function of NK cells and CD8⁺ T cells (Gonzalez et al., 2018[60]). TGF-β also reshapes adaptive immunity by suppressing the differentiation of effector CD4⁺ T-cell subsets while promoting the generation of regulatory T cells, thereby reinforcing immune tolerance. In cytotoxic T cells, TGF-β reduces proliferation and effector cytokine production and promotes the expression of inhibitory receptors associated with T-cell exhaustion, further weakening antitumor responses. In parallel, TGF-β regulates the behavior of myeloid cells throughout tumor progression. During early stages, it influences myeloid differentiation and inflammatory signaling, whereas in advanced tumors, myeloid cells themselves become important sources of TGF-β and matrix-remodeling enzymes, creating a feed-forward loop that sustains immunosuppression and facilitates metastasis (Yang et al., 2008[187]). Genetic or pharmacological disruption of TGF-β signaling in myeloid cells enhances antitumor activity in experimental models, underscoring the central role of this pathway in immune evasion (Novitskiy et al., 2012[128]; Pang et al., 2013[131]). TGF-β not merely as a suppressor of individual immune cell functions but as a key organizer of the immunosuppressive tumor ecosystem, coordinating interactions between stromal, myeloid, and lymphoid compartments to promote tumor progression.

Collectively, these multifaceted effects illustrate how TGF-β signaling operates not only as a regulator of tumor cell behavior but also as a central organizer of the tumor microenvironment, integrating signals that control proliferation, invasion, stromal remodeling, angiogenesis, and immune suppression. This dual role underlies both the therapeutic appeal and the complexity of targeting TGF-β signaling in cancer.

## Therapeutic Strategies Targeting TGF-β Pathway

Therapeutic targeting of the TGF-β pathway has been investigated for more than two decades because of its roles in tumor progression, metastasis, and suppression of antitumor immune responses (Liu et al., 2021[112]; Deng et al., 2024[45]). Early drug development focused on ligand neutralization and inhibition of receptor serine/threonine kinase activity, leading to the development of neutralizing antibodies, ligand traps, receptor-directed antibodies, and small-molecule kinase inhibitors (Cecerska-Heryc et al., 2025[20]). Clinical translation has been limited by dose-related toxicities, insufficient selectivity, and context-dependent signaling responses that complicate patient selection and treatment optimization (Connolly et al., 2012[41]; Katz et al., 2013[87]). These limitations have shifted current strategies toward approaches that modulate receptor trafficking, subcellular localization, and post-translational regulation to achieve more selective pathway control (Di Guglielmo et al., 2003[47]; Chen, 2009[30]). In addition, epigenetic regulators, and non-coding nucleic acid-based interventions have identified additional levels of TGF-β pathway regulation at transcriptional and post-transcriptional stages, expanding potential therapeutic targets beyond receptors and kinases (Papoutsoglou and Moustakas, 2020[132]; Naik and Thakur, 2024[125]). Current development increasingly relies on mechanism-based drug design, biomarker-guided patient selection, and combination therapies to improve clinical efficacy.

### Direct targeting of TGF-β signaling components

#### Receptor kinase inhibitors

TGF-β receptor kinase inhibitors are the most mature small-molecule approach to block TGF-β signaling in cancer and are largely ATP-competitive TβRI inhibitors, often with ancillary activity on ALK4/7. By occupying the ATP-binding pocket of the kinase domain, they prevent ligand-induced SMAD2/3 phosphorylation, suppress SMAD-dependent transcription, and dampen non-canonical pathways such as PI3K-AKT, p38/JNK and NF-κB, leading in preclinical models to reversal of TGF-β-induced EMT, reduced cancer-associated fibroblast activation and matrix deposition, and relief of immune exclusion. Because this inhibitor class now encompasses many chemically distinct scaffolds, the text highlights compounds with the most compelling preclinical and clinical prospects, while additional inhibitors are summarized in Table 1(Tab. 1) (References in Table 1: Callahan et al., 2002[15]; Choi et al., 2023[36]; Choi et al., 2024[37]; DaCosta Byfield et al., 2004[42]; de Gouville and Huet, 2006[44]; Ehata et al., 2007[51]; Faivre et al., 2019[54]; Gellibert et al., 2009[58]; Grygielko et al., 2005[62]; Halder et al., 2005[67]; Kelley et al., 2019[88]; Melisi et al., 2008[119]; Mohammad et al., 2011[120]; Moon et al., 2006[121]; Park et al., 2011[133]; Park et al., 2011[135]; Sawyer et al., 2003[151]; Suzuki et al., 2007[169]; Tojo et al., 2005[173]; Tu et al., 2019[175]; Yap et al., 2021[190]; Yap et al., 2024[188]). Early “tool” compounds such as SB-431542, a prototypical ALK5/ALK4/ALK7 inhibitor, and SD-208, a more drug-like TβRI inhibitor that reduces osteolytic melanoma bone metastases and preserves bone integrity, established that pharmacologic ALK5 blockade can reprogram TGF-β-dependent invasion and metastatic niche formation (Halder et al., 2005[67]; Mohammad et al., 2011[120]), but also revealed scaffold-specific toxicities, including cardiac valvulopathy in preclinical species, driving subsequent efforts toward more selective and carefully dosed agents (Park et al., 2014[134]).

Among clinically advanced TβRI inhibitors, three oral agents illustrate the evolution of this class from monotherapy cytotoxics to microenvironment-modulating combination partners. Galunisertib (LY2157299) is a first-generation, selective ALK5 inhibitor optimized for intermittent dosing (typically 14 days on/14 days off) to mitigate the cardiotoxicity seen with earlier scaffolds (Faivre et al., 2019[54]); in phase II HCC it achieved median overall survival of ~7-17 months as second-line monotherapy with manageable toxicity and pharmacodynamic SMAD2 suppression, and in combination with sorafenib extended overall survival to ~18 months, with additional studies in glioblastoma, pancreatic cancer and nivolumab-based regimens supporting a shift toward rational doublets (Faivre et al., 2019[54]; Kelley et al., 2019[88]). Vactosertib (TEW-7197) represents a newer, highly selective nanomolar ALK5 inhibitor developed to modulate TGF-β-mediated tumor-immune and stromal interactions. In myeloma and solid tumor models it suppresses SMAD2/3 signaling, disrupts tumor-stroma crosstalk and osteolytic disease, and in osteosarcoma downregulates c-MYC and reshapes the microenvironment (↑IFNγ⁺CD8⁺ T cells and NK cells, ↓M2-like macrophages and MDSCs); clinically, early-phase trials combining vactosertib with pembrolizumab in microsatellite-stable metastatic colorectal cancer and other solid tumors have shown antitumor activity and acceptable safety, and ongoing studies pair it with checkpoint inhibitors or chemotherapy to improve responses in tumors characterized by stromal barriers and limited immune infiltration (Park et al., 2014[134]; Choi et al., 2023[36], 2024[37]). LY3200882, a next-generation adenosine analog-based TβRI inhibitor, was designed to further refine selectivity and tolerability; in a first-in-human phase I trial it produced robust pSMAD2 inhibition, no dose-limiting cardiac events, and early signals of efficacy-particularly in pancreatic cancer when combined with gemcitabine/nab-paclitaxel-supporting its continued development in biomarker-enriched cohorts and combination regimens where TβRI blockade is used primarily to remodel the TGF-β-driven tumor microenvironment rather than as a stand-alone cytotoxic therapy (Yap et al., 2021[190]).

Despite their therapeutic potential, TGF-β receptor kinase inhibitors have several limitations. Broad inhibition of TGF-β signaling may disrupt physiological functions such as tissue repair, immune regulation, and vascular homeostasis, raising concerns regarding long-term treatment. Many inhibitors also retain activity against related kinases including ALK4 and ALK7, which may contribute to unintended systemic effects. Preclinical evidence of cardiac valvulopathy associated with continuous ALK5 inhibition has led to intermittent dosing strategies, exposure control, and cardiac monitoring in clinical studies, with current approaches favoring partial and context-dependent pathway modulation rather than sustained complete blockade (Faivre et al., 2019[54]; Guo et al., 2023[66]). In addition, because TGF-β can function as a tumor suppressor during early carcinogenesis, broad inhibition may produce variable therapeutic outcomes depending on tumor context, emphasizing the need for biomarker-based patient selection, particularly in immune-excluded or stroma-rich tumors (Park et al., 2014[134]; Choi et al., 2024[37]).

These considerations have shifted the clinical use of TβRI inhibitors toward combination strategies rather than monotherapy. They are increasingly evaluated together with multikinase inhibitors such as sorafenib in hepatocellular carcinoma (Faivre et al., 2019[54]; Kelley et al., 2019[88]), chemotherapy in pancreatic and colorectal cancers, and PD-1/PD-L1 blockade in microsatellite-stable colorectal cancer, NSCLC, and other immune-resistant solid tumors (Yap et al., 2021[190]). The rationale is to reduce stromal and immunosuppressive barriers and thereby enhance the efficacy of cytotoxic or immune-based therapies. As more selective compounds such as vactosertib and LY3200882 advance through early clinical testing, together with improved pharmacodynamic biomarkers including pSMAD2 and TGF-β-related gene signatures, small-molecule TGF-β receptor kinase inhibitors are expected to remain key components of rational combination regimens targeting pro-tumorigenic TGF-β signaling while preserving its physiological functions.

#### TGF-β ligand traps and receptor-binding blockade in cancer therapy

**Figure 3:**
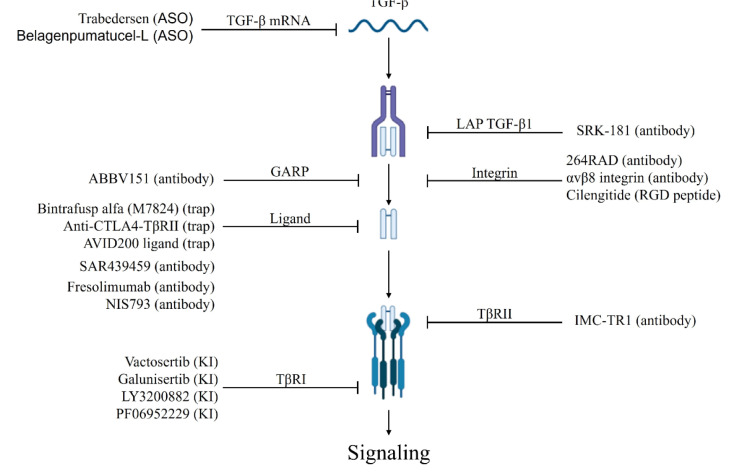
TGF-β Pathway Therapeutic Targets Schematic of TGF-β inhibitors across the pathway, including ASOs targeting TGF-β mRNA, blockade of latent TGF-β activation (LAP, integrins, GARP), ligand neutralization/ sequestration (antibodies, traps), receptor blockade (anti-TβRII), and TβRI/ALK5 kinase inhibitors that prevent downstream signaling.

Extracellular inhibition of TGF-β signaling targets the ligand-receptor interface through strategies that neutralize active ligand, sequester TGF-β using soluble receptor-based traps, or block ligand binding to TGF-β receptors. By preventing ligand interaction with TβRII/TβRI complexes, these approaches reduce SMAD2/3 phosphorylation and downstream gene expression associated with immune suppression, epithelial plasticity, invasion, and stromal remodeling in established tumors. Compared with intracellular kinase inhibitors, extracellular agents can suppress both autocrine and paracrine signaling across multiple tumor microenvironment compartments. Current modalities include neutralizing antibodies against specific TGF-β isoforms, engineered ligand traps with isoform selectivity, and receptor-directed antibodies that interfere with receptor complex formation (Table 2(Tab. 2); References in Table 2: Bauer et al., 2023[7]; Cho et al., 2023[34]; Gulley et al., 2022[63]; Mascarenhas et al., 2023[118]; Morris et al., 2014[122]; Paz-Ares et al., 2020[139]; Shimizu et al., 2024[162]; Tolcher et al., 2017[174]; Welsh et al., 2021[180]; Yap et al., 2026[189]). In addition, activation-selective strategies aim to inhibit local generation of active TGF-β by targeting components of latent TGF-β activation complexes, such as GARP (glycoprotein A repetitions predominant), a cell-surface protein that presents latent TGF-β_1_ and facilitates its activation in specific cellular contexts (Zimmer et al., 2022[195]) (Figure 3(Fig. 3)). This approach seeks to limit pathologic TGF-β signaling while preserving systemic homeostatic functions.

Key translational challenges include the pleiotropic physiological functions of TGF-β, compensatory signaling among TGF-β isoforms and related pathways, and limited intratumoral distribution and pharmacodynamic activity of extracellular inhibitors. As a result, most extracellular TGF-β inhibitors are evaluated in combination with PD-1/PD-L1 blockade and/or cytotoxic therapy rather than as monotherapies. Importantly, safety findings indicate that systemic inhibition of TGF-β signaling produces adverse effects similar to those observed with TGF-β receptor kinase inhibitors, reflecting on-target pathway suppression across normal tissues. For example, early clinical studies of the pan-TGF-β antibody fresolimumab (GC1008) reported dermatologic proliferative lesions consistent with systemic pathway inhibition (NCT00356460) (Morris et al., 2014[122]).

NIS793 is a neutralizing anti-TGF-β antibody that has been evaluated mainly as an immunotherapy partner. A phase I/Ib study tested NIS793 with the anti-PD-1 antibody spartalizumab in advanced solid tumors (NCT02947165), with follow-on phase II evaluation in metastatic pancreatic ductal adenocarcinoma in combination regimens (NCT04390763) (Bauer et al., 2023[7]). These studies reflect the hypothesis that partial relief of TGF-β-associated immune suppression may increase susceptibility to checkpoint blockade in selected tumor contexts.

AVID200 is an engineered trap designed to sequester TGF-β_1_ and TGF-β_3_ while sparing TGF-β_2_; it has been evaluated in a phase I dose-escalation study in advanced/metastatic solid tumors (NCT03834662) and has also been clinically characterized in myelofibrosis (Gulley et al., 2022[63]; Mascarenhas et al., 2023[118]). This approach of isoform-selective trapping has been pursued to modulate efficacy-tolerability balance.

A related but distinct strategy combines TGF-β trapping with immune checkpoint inhibition in a single molecule. Bintrafusp alfa (M7824) couples PD-L1 blockade with a TGF-β trap domain derived from the TβRII ectodomain. Early phase evaluation supported clinical feasibility (e.g., NCT02517398) (Paz-Ares et al., 2020[139]). However, the phase III INTR@PID Lung 037 study in first-line PD-L1-high NSCLC (NCT03631706) did not show superiority over pembrolizumab and was terminated for futility, emphasizing that combined targeting does not necessarily translate into benefit without appropriate biological selection and treatment context (Cho et al., 2023[34]). Direct receptor blockade has also been evaluated clinically. LY3022859 (IMC-TR1), an anti-TGFβRII antibody, was tested in a first-in-human phase I study in advanced solid tumors (NCT01646203). Higher doses were limited by cytokine release-type toxicity, and a maximum tolerated dose was not established (Tolcher et al., 2017[174]). These findings indicate tolerability limitations associated with receptor-directed blockade at the cell surface.

To increase selectivity for tumor-relevant TGF-β activity, activation-selective programs have advanced in parallel. SRK-181 (linavonkibart) is an antibody that binds latent TGF-β_1_ and inhibits activation; it is being evaluated clinically in advanced solid tumors, including combination with pembrolizumab (NCT04291079) (Welsh et al., 2021[180]; Yap et al., 2026[189]). Livmoniplimab (ABBV-151) targets the GARP:TGF-β1 complex to prevent activation of latent TGF-β_1_ and is in phase I development as monotherapy and in combinations (NCT03821935) (Shimizu et al., 2024[162]). These approaches are intended to suppress localized activation within the tumor microenvironment while reducing the likelihood of systemic toxicities associated with broad neutralization.

Overall, ligand traps and receptor-binding inhibitors provide mechanistically direct methods to suppress extracellular TGF-β signaling, with clinical programs increasingly emphasizing combination regimens and, in some cases, activation-selective targeting. Further progress is likely to depend on aligning the inhibitory format (pan-ligand, isoform-selective trap, activation-selective, or receptor-directed) with the dominant source and mode of TGF-β signaling in a given tumor context and on incorporating pharmacodynamic and microenvironmental biomarkers to guide patient selection and combination design.

### Modulation of TGF-β receptor trafficking and localization

#### Membrane distribution and compartmentalization of TGF-β receptors

**Figure 4:**
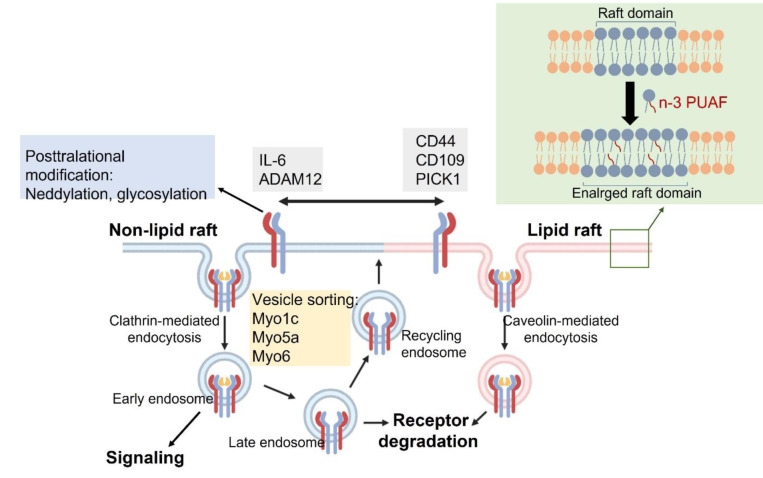
Membrane distribution of TGF-β receptors is regulated by multiple mechanism TGF-β receptors partition between raft (red) and non-raft (blue) domains. Caveolin-mediated uptake promotes degradation, whereas clathrin-mediated uptake supports signaling and receptor recycling. Receptors exchange between domains, and distribution is regulated by co-receptors/membrane partners, lipid composition, trafficking/motor proteins, and receptor post-translational modifications, including altered glycosylation.

The magnitude and duration of TGF-β signaling are not determined solely by ligand availability or receptor expression, but are also critically influenced by the spatial distribution of receptors within the plasma membrane and their subsequent intracellular trafficking. The density of TGF-β receptors at the cell surface directly affects signaling input, whereas their partitioning into distinct membrane microdomains can differentially regulate downstream signaling outcomes. Receptors internalized through clathrin-mediated pathways are typically associated with signaling propagation and recycling, whereas localization within lipid raft or caveolae domains is frequently linked to receptor turnover and attenuation of signaling (Huang and Chen, 2012[78]) (Figure 4(Fig. 4)). Thus, the balance between these internalization routes represents a key determinant of signaling intensity and duration, and defects in receptor localization or trafficking can impair pathway activity even when total receptor levels remain unchanged (Capocasale et al., 1995[19]). 

Accumulating evidence indicates that membrane lipid composition and microdomain organization play central roles in regulating TGF-β receptor compartmentalization and signaling. Lipid rafts, which are highly ordered membrane microdomains enriched in cholesterol, sphingolipids, and saturated phospholipids, provide specialized platforms that organize receptor interactions and influence signaling efficiency (Brown and London, 2000[13]). The size, composition, and mobility of these microdomains are critical determinants of their function, as incorporation or extraction of specific lipid components can reshape raft structure and alter raft-mediated cellular responses. Cholesterol, a key structural component maintaining raft stability, has emerged as an important regulator of TGF-β responsiveness by controlling receptor localization within the plasma membrane. Experimental depletion of cholesterol using agents such as nystatin or methyl-β-cyclodextrin shifts TβRII toward non-raft regions, whereas elevated cholesterol promotes accumulation of TGF-β receptors within lipid raft/caveolae domains, resulting in reduced SMAD activation and attenuation of downstream signaling (Di Guglielmo et al., 2003[47]; Chen et al., 2008[25]). These findings suggest that cholesterol-rich rafts can function as compartments that sequester receptors and favor receptor turnover, thereby limiting signaling output.

Beyond cholesterol, alterations in membrane lipid composition can further modulate raft organization and receptor partitioning. n-3 polyunsaturated fatty acids (PUFAs), particularly eicosapentaenoic acid (EPA) and docosahexaenoic acid (DHA), have been shown to alter lipid raft architecture by inducing phase separation between cholesterol-rich and DHA-enriched membrane domains (Shaikh et al., 2003[159]; Stillwell and Wassall, 2003[167]; Stillwell et al., 2005[166]) (Figure 4(Fig. 4)). Studies showed that DHA exerts anti-cancer effect by reducing expression of raft-associated onco-proteins including EGFR and Hsp90 through inducing raft fusion with lysosomes in breast cancer (Rogers et al., 2010[149]; Lee et al., 2014[99]). Also, the role of DHA on T cell activation was determined and found that dietary DHA altered lipid raft partitioning of key protein involved in T cell activation. Recruitment of PKCθ into lipid rafts was suppressed by DHA, causing downregulation of the following signaling and inhibition in lymphoproliferation (Fan et al., 2004[55]). Several studies demonstrated the inhibitory effect of DHA on TGF-β responses. DHA has been shown to prevent TGF-β-induced myofibroblast differentiation, invasiveness and EMT in human prostate cancer as well as inhibiting angiogenesis through inhibiting TGF-β-induced endothelial cell migration (Bianchini et al., 2012[10]; Guo et al., 2021[64]). Although the precise mechanisms remain incompletely defined, these observations raise the possibility that DHA-mediated modification of raft microenvironments alters the membrane distribution of TGF-β receptors, potentially move receptor trafficking toward degradation pathways. Importantly, partitioning of proteins into lipid rafts does not uniformly enhance signaling; depending on raft organization, it may either facilitate signaling through increased protein-protein interactions or suppress signaling through enhanced endocytosis and turnover. To maintain optimal bioactivity, lipid rafts must remain small and dynamic, typically within a diameter range of 6-14 nm (Nicolau Jr et al., 2006[127]). Studies comparing T cells from wild-type and fat-1 transgenic mice, which endogenously produce n-3 PUFA, showed that n-3 PUFA enlarge lipid raft domains, likely due to reduced affinity between n-3 PUFA and cholesterol- or sphingolipid-rich regions, resulting in impaired raft function and reduced T-cell activation (Kim et al., 2008[91]). Similar enlargement and clustering of lipid rafts induced by n-3 PUFA have also been observed in HeLa cells (Chapkin et al., 2008[21]). Together, these findings support the concept that n-3 PUFAs possess anti-inflammatory and anti-cancer potential through modulation of raft architecture. However, despite the clear effects of DHA on TGF-β responses, direct evidence demonstrating that n-3 PUFAs regulate membrane distribution of TGF-β receptors remains lacking, highlighting an important area for future investigation.

Pharmacologic and naturally occurring compounds have further illustrated the functional importance of receptor compartmentalization. Euphol, a triterpene structurally similar to cholesterol, suppresses TGF-β signaling by promoting the segregation of TGF-β receptors into lipid raft microdomains, facilitating receptor degradation and reducing SMAD activation (Chen et al., 2015[23]). Similarly, oxidized cholesterol derivatives such as cholest-4-en-3-one attenuate TGF-β signaling by driving the translocation of receptors into lipid raft domains and accelerating their degradation (Chen et al., 2017[26]; Huang et al., 2017[80]). In contrast, betulinic acid has been shown to enhance TGF-β responsiveness by shifting receptors from lipid raft/caveolae regions to non-raft microdomains, thereby favoring signaling-competent endosomal pathways (Chen et al., 2016[22]). These observations collectively indicate that altering receptor partitioning between membrane compartments can either suppress or potentiate TGF-β signaling without necessarily changing total receptor levels.

In addition to lipid composition, extracellular matrix components can regulate receptor compartmentalization through interactions with membrane proteins (Figure 4(Fig. 4)). Hyaluronan (HA), a major component of the extracellular matrix, has been shown to modulate TGF-β receptor localization through its interaction with CD44. Binding of HA with CD44 promotes MAP kinase-dependent redistribution of TGF-β receptors into caveolin-associated lipid raft compartments, facilitating receptor turnover and attenuating SMAD signaling, whereas disruption of HA-CD44 interaction prevents this redistribution, highlighting the importance of receptor-matrix interactions in controlling receptor trafficking (Ito et al., 2004[81]). This HA-dependent signaling environment can also influence receptor activation and cytoskeletal organization, as inflammatory stimuli are known to enhance HA production and CD44 expression, thereby promoting the formation of HA-CD44-ERM protein complexes that associate with TGF-β receptors and cytoskeletal microdomains, modulating receptor localization and downstream cellular responses (Takahashi et al., 2010[171]). Collectively, these findings indicate that receptor compartmentalization is not governed solely by membrane lipid composition but is dynamically regulated by extracellular matrix organization, receptor-cytoskeleton interactions, and microenvironmental cues that together regulate TGF-β signaling.

Clinically used agents may also influence receptor compartmentalization. Sorafenib, an approved multikinase inhibitor for hepatocellular carcinoma, suppresses TGF-β responsiveness primarily by accelerating TβRII turnover through a membrane domain-dependent mechanism rather than direct inhibition of receptor kinase activity. Sorafenib promotes caveolae-associated internalization and lysosome-dependent degradation of TβRII, and in epithelial-like cells this process is accompanied by recruitment of receptors from non-raft regions into caveolin-positive domains, thereby sustaining receptor depletion across membrane compartments. Notably, this effect is cell-type dependent: in hepatic stellate cells, the non-raft receptor pool appears less mobile, limiting inter-domain redistribution and rendering these cells relatively resistant to sorafenib-induced receptor loss and SMAD inhibition. These observations suggest that membrane microdomain dynamics and receptor mobility can critically influence the therapeutic impact of agents that target receptor trafficking (Chung et al., 2018[40]).

Collectively, these observations indicate that membrane distribution and compartmentalization represent a critical regulatory layer of TGF-β signaling, integrating inputs from lipid composition, extracellular matrix remodeling, and signaling adaptor proteins. Because internalized receptors must subsequently be transported between endosomal compartments and other intracellular destinations, these processes are closely linked to the mechanisms that control vesicle trafficking and motor protein-mediated transport, which are discussed in the following section.

#### Intracellular vesicle trafficking and motor proteins

Internalization and intracellular trafficking of lipid raft are actin- and microtubule-dependent processes, and motor proteins such as myosin, kinesin, and dynein play essential roles in those processes. Through converting the chemical energy from ATP hydrolysis, myosin protein is the motor molecule which has been known to move along actin filament with their tail carrying cargo such as organelle and intracellular vesicle (Pollard et al., 1974[144]; Balasubramanian et al., 2007[3]; Hammer and Sellers, 2012[68]). Myosin 1c has been shown to regulate intracellular distribution and the recycling process of cargo associated with lipid raft domains (Brandstaetter et al., 2012[12]). Our study demonstrated that pentachloropseudilin (PClP), a reversible and allosteric inhibitor of Myo1c, inhibited recycling of TβRII to the cell surface, resulting in accumulation of TβRII in the late endosomes and recycling endosomes, and eventually those accumulated TβRII were sorted to lysosome for degradation. Thereby, PClP attenuated TGF-β/SMAD signaling (Chinthalapudi et al., 2011[33]; Chung et al., 2018[39]). The similar results are observed from our study in pentabromopseudilin (PBrP), an inhibitor of myosin Va (MyoVa). We found that PBrP and gene deletion of MyoVa attenuated TGF-β/SMAD signaling and TGF-β-induced fibronectin, PAI-1, and EMT protein expression through facilitating turnover of cell surface TβRII. Inhibition and deletion of MyoVa promote cell surface TβRII internalized in lipid-raft membrane fractions which has been known to sort the TGF-β receptor for degradation (Shih-Wei et al., 2018[161]). MyoVa moves a broad range of receptors from cytoplasm to the plasma membrane, thereby regulating their activities and downstream signaling. For instance, MyoVa has been reported to transport glutamate receptors and GLUT4 (Lisé et al., 2006[107]; Sun et al., 2014[168]). Myosin VI (Myo6), unlike other myosin, is the only known myosin that moves toward the minus end of actin filaments and mediates multiple transport steps (Wells et al., 1999[179]). Myo6 has been identified to localize into clathrin-coated pits and drive clathrin-mediatd endocytosis (Buss et al., 2001[14]). Morris et al. found that Myo6 links to clathrin-coated pits through binding of the adaptor protein Dab2, which then binds to clathrin adaptor protein AP-2. They hypothesized that the Myo6-Dab2 complex may serve as the link between the actin cytoskeleton and receptor endocytosis (Morris et al., 2002[123]). Dab2 is required for TGF-β-induced responsiveness such as EMT and migration, and loss of Dab2 impaired recycling of TβRII and TβRI (Hocevar et al., 2005[75]; Prunier and Howe, 2005[147]; Penheiter et al., 2010[140]). Hence, Myo6 might play roles in intracellular and membrane trafficking of TGF-β receptors, possibly mediated through interacting with Dab2. Considering that most of the TGF-β receptors localize intracellularly, molecules which control intracellular vesicle trafficking or sorting are key mediators and could develop as novel molecular targets in treatment of TGF-β-related diseases.

#### Post-translational modifications controlling receptor fate

Both TβRI and TβRII are known to be modified by multiple molecules, and the modification of TGF-β receptors is highly associated with receptor functions. TGF-β receptors could be modified by glycosylation, phosphorylation, neddylation, sumoylation, and ubiquitination. It is well established that phosphorylation of the receptor triggers signaling transduction and ubiquitination results in proteasomal degradation of TGF-β receptors. Neddylation and glycosylation of TGF-β receptors are demonstrated to impact signaling transduction through targeting membrane distribution of the receptors. Casitas B-lineage lymphoma (c-Cbl), a proto-oncogene, function as a NEDD8 E3 ligase which triggered neddylation of TβRII, and the addition of NEDD8 molecule further stabilized TβRII in non-raft domains and prevented receptor from being internalized through caveolin-mediated endocytosis (Zuo et al., 2013[197]). Pharmacological modulation of post-translational modifications further supports their functional importance in regulating TGF-β signaling. Inhibition of neddylation by MLN4924 (pevonedistat), a selective NEDD8-activating enzyme inhibitor, has been shown to suppress pathological fibrosis by attenuating TGF-β-associated signaling responses, highlighting the translational potential of targeting PTM machinery to modulate TGF-β activity (Shen et al., 2026[160]). Along a related axis, recent studies demonstrate that SUMOylation of SMAD4 represents a critical regulatory node controlling TGF-β-driven fibrotic signaling. Ginkgolic acid (GA), a pharmacological inhibitor of SUMOylation, alleviates infection-induced hepatic fibrosis by suppressing SMAD4 SUMOylation and limiting its nuclear translocation, thereby reducing TGF-β-dependent transcriptional activity and disrupting profibrotic macrophage-hepatic stellate cell crosstalk. GA treatment was further associated with reduced TGF-β_1_ levels and attenuation of fibrosis markers *in vivo*, supporting a mechanistic link between SUMOylation control and TGF-β signaling output (Chen et al., 2026[28]). Glycosylation represents another important regulatory layer controlling receptor localization and signaling competence. Studies have demonstrated that suppression of N-linked glycosylation of TβRII disrupts both intracellular trafficking and membrane partitioning of the receptor. Systemic inhibition of glycosylation using tunicamycin or genetic deletion of Mgat5 (Alpha-1,6-Mannosylglycoprotein 6-Beta-N-Acetylglucosaminyltransferase) perturbed raft partitioning of TβRII and shifted receptor into non-raft domains (Luga et al., 2009[114]). Further mechanistic analysis suggested that this redistribution may result from altered interactions between TβRII and other membrane glycoproteins, as TβRII was shown to associate with galectin-3 in an Mgat5-dependent manner in transgenic mouse models (Partridge et al., 2004[136]). Our recent study showed that global perturbation of glycosylation by using prodigiosin, a marine-derived compound, inhibited TGF-β induced SMAD pathway and response in human lung cancer and liver cancer cells. Treatment of prodigiosin resulted in sequestration of non-glycosylated or immature TβRII in the perinuclear region with inhibition in intracellular vesicle trafficking of mature TβRII, thereby leading to signaling turn over (Tai et al., 2024[170]). 

#### Galectin-targeted inhibitors as indirect modulators of TGF-β receptor signaling

Galectins are glycan-binding “readers” that recognize β-galactoside-containing glycans (e.g., N-acetyllactosamine) on cell-surface glycoproteins and can regulate receptor organization and signaling. In cancer, elevated galectin production frequently associates with worse clinical outcomes and treatment resistance, motivating efforts to pharmacologically inhibit galectin-glycan interactions (Laderach and Compagno, 2022[96]). High galectin-3 expression has been linked to aggressive behavior and poor clinical outcomes in NSCLC and primary HCC. In NSCLC cohorts, tumor galectin-3 expression associates with inferior survival metrics (Kusuhara et al., 2021[95]), while in HCC, genetic depletion of galectin-3 suppresses proliferation, migration, and invasion and promotes apoptosis in vitro (Jiang et al., 2014[84]). Extracellular galectin-3 can oligomerize and crosslink glycoproteins to form a “galectin lattice,” which stabilizes TGF-β receptors at the cell surface, limits their internalization, and thereby increases receptor abundance available for ligand stimulation, resulting in enhanced downstream signaling.

Building on these observations, several classes of galectin antagonists have been used to reduce TGF-β signaling activity by disrupting glycan-dependent interactions that sustain receptor availability and productive signaling at the plasma membrane (Table 3(Tab. 3)). A well-characterized class comprises small-molecule galectin-3 carbohydrate recognition domain (CRD) antagonists, which competitively inhibit galectin-3 binding to β-galactoside-containing glycans and thereby disrupt extracellular lattice formation. In human lung fibroblasts, a recent study showed that extracellular galectin-3 not only organizes surface glycoproteins but can also couple more directly to the TGF-β machinery by engaging αv integrins and glycosylated TβRII; in this setting, pharmacologic galectin-3 inhibition with GB0139/TD139 suppressed SMAD2 phosphorylation, consistent with diminished receptor-proximal signaling (Calver et al., 2024[17]). Notably, GB0139 was originally optimized for pulmonary delivery in fibrotic lung disease, providing a translationally relevant example of how targeting extracellular galectin-3 can restrain TGF-β-driven processes without directly inhibiting the receptor kinase domain (Hirani et al., 2021[74]). In parallel, orally bioavailable galectin-3 inhibitors such as GB1107 and GB1211 (selvigaltin)-which has progressed into clinical-stage development; these agents are generally positioned as CRD antagonists that interfere with galectin-3-glycan binding, and have been applied in oncology models where suppression of pro-tumorigenic phenotypes (including invasion, stromal remodeling, and therapy resistance) plausibly intersects with reduced TGF-β-dependent EMT and microenvironmental cues (Calver et al., 2024[17]).

In addition to Galectin-3, several inhibitors targeting other galectins have been applied in contexts where attenuation of TGF-β signaling is mechanistically plausible or has been directly observed. Thiodigalactoside (TDG), a widely used β-galactoside mimetic, functions as a broad CRD competitor across multiple galectins and is deployed as a chemical tool to disrupt galectin-glycan interactions. TDG has been used to probe whether TGF-β-dependent phenotypes require extracellular lectin-glycan engagement rather than receptor kinase activity per se. OTX008 (PTX008), by contrast, is typically described as a Galectin-1-targeting small molecule and has been applied in contexts where Galectin-1 contributes to EMT-like remodeling and fibrotic responses. Notably, Galectin-1 blockade has been linked to decreased expression of TGF-β_1_ and TGF-β receptors-supporting the notion that Galectin-1 inhibition may reduce TGF-β signaling both by limiting extracellular glycan-mediated receptor organization and by lowering pathway availability at the ligand/receptor expression level (Xue and Li, 2023[185]; Balta et al., 2024[4]). Neutralizing anti-Galectin-9 (Gal-9) antibodies are primarily used to modulate immune responses, but they can also influence the TGF-β axis via immunosuppressive pathways. Gal-9 has been reported to promote regulatory T cell (Treg) differentiation and/or function, potentially in a TGF-β-dependent manner. Thus, anti-Gal-9 therapy may indirectly weaken TGF-β-supported immunosuppressive niches in the tumor microenvironment, even when canonical tumor-cell SMAD signaling is not the main target (Lv et al., 2013[116]). In addition to these defined galectins, multivalent carbohydrate-based galectin antagonists-including modified citrus pectin (MCP) and related modified pectin preparations, as well as polysaccharide formulations such as belapectin (GR-MD-02), GM-CT-01 (davanat), and GCS-100-have been widely used as functional galectin inhibitors in preclinical studies. Although these macromolecular agents can vary in composition and target selectivity, their shared principle is to sequester or competitively engage galectins through multivalent glycan presentation, thereby weakening lattice assembly and the associated stabilization of receptor-rich surface domains. 

These pharmacological studies raise a key mechanistic question: does galectin boost TGF-β signaling mainly by binding glycans on TβRII itself, or by clustering TβRII with other surface glycoproteins that control raft/caveolae localization, endocytic trafficking, and integrin crosstalk? Dissecting these alternatives-particularly the relative contributions of receptor-intrinsic glycans versus neighboring glycoprotein networks-will be crucial for explaining context specificity (e.g., why galectin inhibition strongly suppresses TGF-β outputs in certain stromal or EMT-prone niches but not others). This distinction will also help determine which inhibition approach-high-affinity CRD antagonists or multivalent glycan mimetics-is most likely to suppress TGF-β-driven oncogenic programs in tumors with specific glyco-phenotypes.

#### Nuclear shuttling and signal termination

Nucleocytoplasmic shuttling is a key control point in TGF-β signaling. SMAD proteins continually move between the cytoplasm and nucleus, and pathway output depends on the balance of their nuclear import and export. SMAD export is mediated by CRM1; blocking CRM1 (for example with leptomycin B) causes SMADs to accumulate in the nucleus and changes signaling dynamics (Pierreux et al., 2000[142]). CRM1 also exports negative regulators such as Smurf1 and SMAD7, which helps terminate TGF-β signaling (Hata and Chen, 2016[69]). Import is equally important: activated SMADs enter the nucleus via an importin-β-dependent mechanism, and disrupting this transport attenuates TGF-β-driven transcription. Together, these findings support nucleocytoplasmic transport as a practical, indirect way to tune TGF-β signaling, small molecules that disrupt import or export-such as importazole, which inhibits importin α/β-mediated transport-show that SMAD trafficking can be targeted pharmacologically and may complement other approaches to inhibit TGF-β-dependent transcriptional programs (Soderholm et al., 2011[164]).

### Indirect and contextual modulation of TGF-β signaling

#### Regulation of type III TGF-β receptor and co-receptors

**Figure 5:**
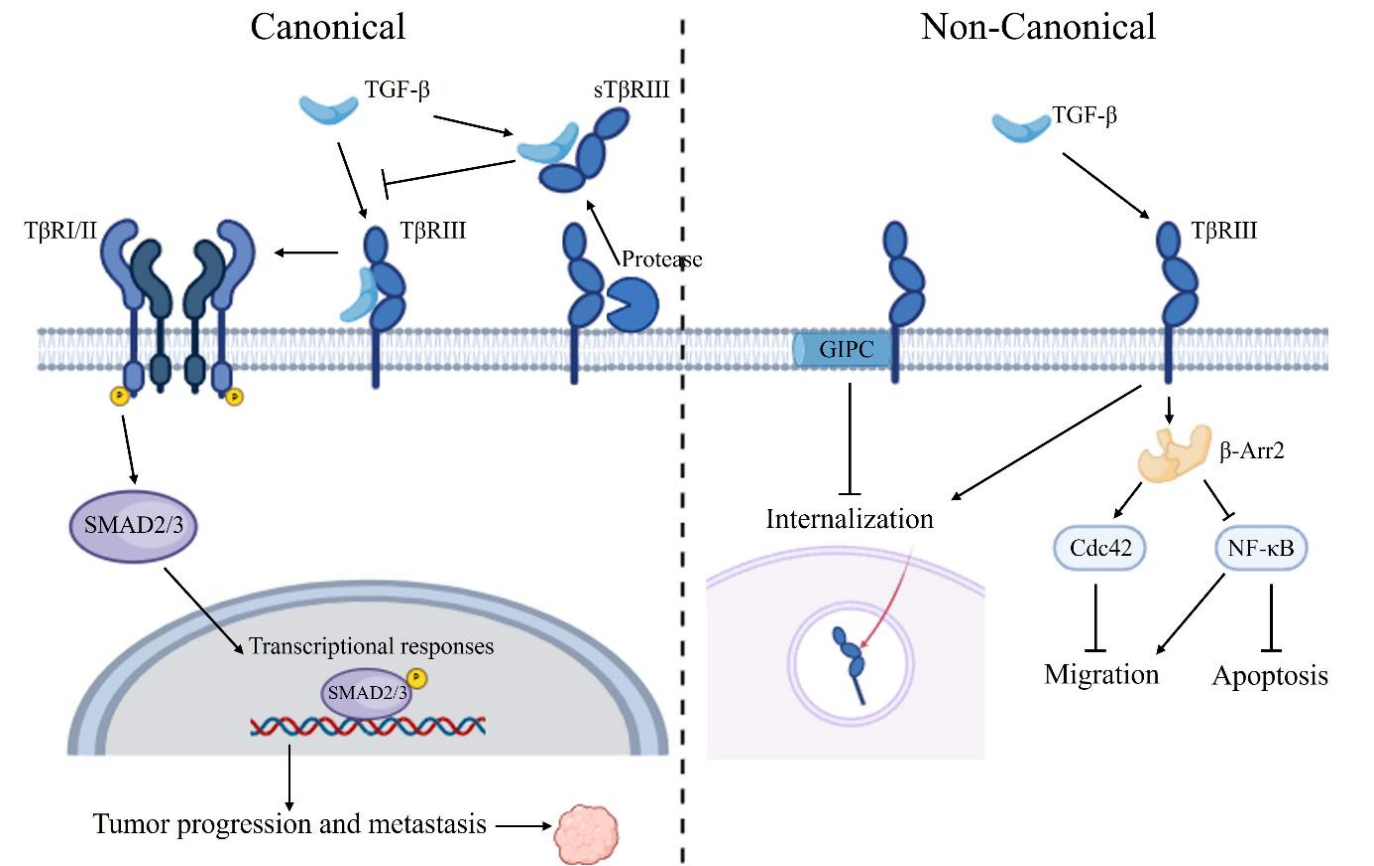
Betaglycan (TβRIII) Modulates TGF-β Signaling Schematic of TβRIII (betaglycan) regulation of TGF-β signaling. Canonical: membrane and soluble TβRIII sequester ligand and limit SMAD2/3 activation, reducing transcriptional responses linked to tumor progression. Non-canonical: TβRIII interacts with GIPC/β-arrestin2 to regulate internalization, Cdc42 and NF-κB, affecting migration and apoptosis.

In addition to the TβRI and TβRII that mediate canonical TGF-β signaling, accessory co-receptors play important roles in regulating ligand availability, receptor complex formation, trafficking, and signaling output. Earlier reviews by Blobe and colleagues highlighted that these co-receptors provide critical additional layers of control over TGF-β signaling and contribute to context-dependent cellular responses. Subsequent studies have expanded this framework and identified multiple co-receptors-including type III TGF-β (TβR3 or betaglycan), endoglin (CD105), CD109, BAMBI, neuropilins, Cripto-1, and repulsive guidance molecules (RGMs)-that modulate signaling through mechanisms such as ligand sequestration, receptor internalization, and pathway cross-talk. These co-receptors have been implicated in key cancer-related processes, including angiogenesis, epithelial-mesenchymal transition, migration, and metastasis, underscoring their importance in tumor progression and therapeutic response (Pawlak and Blobe, 2022[138]). Among these molecules, betaglycan has attracted particular interest because of its tumor-suppressive properties and ability to regulate ligand availability. Reduced expression of betaglycan is frequently observed in tumor tissues compared with adjacent normal epithelium, and diminished betaglycan levels have been associated with poorer clinical outcomes, suggesting a tumor-suppressive role in cancer progression (Hempel et al., 2007[72]; Listik et al., 2021[108]). Betaglycan regulates tumor cell behavior through both TGF-β-dependent and TGF-β-independent mechanisms (Figure 5(Fig. 5)). In its soluble form, betaglycan can attenuate TGF-β signaling by sequestering ligands and limiting their interaction with signaling receptors, thereby reducing downstream SMAD activation and suppressing tumor cell migration and invasion (Dong et al., 2007[48]; Elderbroom et al., 2014[52]; Choi et al., 2024[35]). In addition to these ligand-sequestering effects, betaglycan has also been reported to inhibit cell invasion independently of TGF-β signaling, partly through activation of the p38 pathway and β-arrestin2-mediated activation of Cdc42, which influence cytoskeletal dynamics and cell motility (Santander and Brandan, 2006[150]; Mythreye and Blobe, 2009[124]; Lee et al., 2010[100]). Collectively, these findings suggest that therapeutic strategies aimed at increasing betaglycan levels-either by enhancing its expression or modulating its processing-may represent an underappreciated approach to limit cancer invasion.

Recent evidence indicates that pharmacological induction of betaglycan is feasible through distinct signaling pathways. Our current mechanistic studies demonstrate that fluoroquinolones, particularly ciprofloxacin, suppress cancer cell migration and metastasis through activation of a cAMP-Epac (RAPGEF3) signaling axis that promotes betaglycan expression (Liu et al., 2025[110]). This Epac-dependent upregulation of betaglycan establishes a previously unrecognized link between fluoroquinolone signaling and modulation of TGF-β co-receptor composition, thereby shifting cellular signaling toward a less invasive phenotype. In parallel, fluoroquinolones have also been shown to induce IGFBP-3 expression in a p53-dependent manner, resulting in growth inhibition through both IGF-dependent and IGF-independent pathways (Chung and Chen, 2024[38]). Given the known crosstalk between IGFBP-3 and TGF-β-related signaling networks, fluoroquinolone-induced IGFBP-3 may act synergistically with betaglycan upregulation to reinforce anti-proliferative and anti-invasive cellular states, further supporting the concept that fluoroquinolones function as multi-layered modulators of tumor-suppressive signaling programs.

Notably, earlier studies demonstrated that glucocorticoids such as dexamethasone selectively enhance betaglycan expression through glucocorticoid receptor-dependent transcriptional mechanisms in osteoblast-like cells and hepatic stellate cells, without comparable upregulation of type TβRI and TβRII (Nakayama et al., 1994[126]; Wickert et al., 2004[181]). These findings collectively support a broader concept whereby pharmacological agents can fine-tune TGF-β signaling not only by targeting ligands or kinase activity directly, but also by altering co-receptor availability. In addition, glucocorticoid and fluoroquinolone-induced betaglycan may additionally favor its shedding and generation of soluble betaglycan (sBG), which could function as a ligand trap to sequester TGF-β and further attenuate downstream signaling, suggesting that fluoroquinolones may regulate TGF-β activity at multiple levels, including receptor abundance and ligand availability.

Other co-receptors, such as endoglin, CD109, BAMBI, neuropilins, and Cripto-1, also influence signaling intensity or receptor trafficking and may represent additional targets for therapeutic modulation, although their translational potential remains less fully explored.

#### AMPK agonists restrain TGF-β in cancer

AMP-activated protein kinase (AMPK) is a heterotrimeric serine/threonine kinase that functions as a central metabolic sensor, integrating energetic stress with broad transcriptional and post-translational programs. Beyond its classical role in inhibiting anabolic processes and promoting catabolism, AMPK exerts tumor-suppressive functions by constraining growth factor signaling, mTOR activity, and epithelial-mesenchymal transition (EMT) (Gao et al., 2018[56]). A growing body of work indicates that AMPK negatively regulates TGF-β signaling at several levels, including suppression of TGF-β production, interference with receptor complex activation, and attenuation of SMAD-dependent transcription, thereby providing a mechanistic rationale for targeting the AMPK-TGF-β axis in oncology (Li et al., 2016[104]; Gao et al., 2018[56]; Zou et al., 2021[196]).

Metformin is the best-characterized AMPK activator in this context and provides a prototypical example of drug repurposing (Table 4(Tab. 4); References in Table 4: Ashrafizadeh et al., 2020[2]; Chen et al., 2014[27]; Ge et al., 2019[57]; Jin et al., 2022[85]; Liu et al., 2022[113] ; Wang et al., 2022[178]; Yoshida et al., 2020[193]). In lung adenocarcinoma, prostate, liver, and pancreatic cancer models, metformin-induced AMPK activation inhibits TGF-β-stimulated SMAD2/3 phosphorylation and nuclear translocation, leading to suppression of EMT, migration and invasion (Lin et al., 2015[106]; Yoshida et al., 2020[193]; Wang et al., 2021[177]). Mechanistically, metformin has been reported to interfere with TβRII dimerization and downstream SMAD activation, and to reduce TGF-β_1_ mRNA levels and SMAD3-mediated autoinduction in gastric cancer cells (Xiao et al., 2016[184]). In glioblastoma, cervical and other solid tumors, metformin reverses TGF-β-induced EMT-like changes and cancer stem-like features, often via AMPK-dependent inhibition of AKT/mTOR and SMAD pathways (Cheng and Hao, 2016[32]; Song et al., 2018[165]). 

AICAR (5-aminoimidazole-4-carboxamide ribonucleoside), a classic experimental AMPK agonist, extends this concept into stromal and fibrotic models that are highly relevant to tumor biology. In unilateral ureteral obstruction and renal fibroblast systems, AICAR robustly increases AMPK phosphorylation, reduces TGF-β-induced myofibroblast activation, and attenuates expression of collagen and α-smooth muscle actin, in association with reduced SMAD3, ERK1/2 and STAT3 signaling (Chen et al., 2014[27]). Similar AMPK-dependent suppression of TGF-β-driven fibrogenesis has been observed in hepatic stellate cells and other mesenchymal cell types (Lee et al., 2013[101]; Li et al., 2015[103]). Although most of these studies focus on non-malignant fibrosis, the same myofibroblast-like phenotypes and ECM programs operate in cancer-associated fibroblasts, implying that AICAR-like AMPK activators could theoretically normalize tumor stroma and limit TGF-β-driven desmoplasia that supports invasion and therapeutic resistance.

IMM-H007 (WS070117), an adenosine-derived AMPK activator, provides an example of a dual-function small molecule that modulates both AMPK and TGF-β signaling more directly. In β-adrenergic and angiotensin II-driven cardiac fibrosis models, IMM-H007 activates AMPK, decreases TGF-β_1_ expression, and attenuates SMAD2/3 phosphorylation, leading to marked reductions in collagen deposition and myofibroblast markers (Ge et al., 2019[57]; Wang et al., 2022[178]). In addition, biophysical studies indicate that IMM-H007 directly binds TGF-β and disrupts its interaction with TβRII. Although shown in cardiovascular models, these support the concept of AMPK activators that also function as TGF-β antagonists, potentially useful in cancers with TGF-β-driven fibrosis and stiff, pro-invasive stroma.

Natural compounds such as berberine further bridge AMPK activation, TGF-β inhibition, and explicit anticancer effects. In chronic pancreatitis and pancreatic fibrosis models, berberine activates AMPK and, suppresses TGF-β/SMAD signaling and M2 macrophage polarization, thereby mitigating fibrotic remodeling (Bansod et al., 2020[6]). Importantly, several studies have extended these findings to malignancy: berberine inhibits EMT and promotes apoptosis in both normal and cancerous colon epithelial cells via coordinated inhibition of TGF-β1/SMAD and NF-κB p65 signaling, partly through regulation of miR-1269a (Huang et al., 2020[77], 2024[76]). In glioma, liver, and gastric cancer models, berberine reduces migration, invasion and EMT by downregulating TGF-β/SMAD2/3 signaling. Together, these data support berberine as a scaffold for developing AMPK-activating, TGF-β-inhibiting agents with direct anti-metastatic activity (Du et al., 2021[49]; Jin et al., 2022[85]).

Resveratrol, a polyphenolic compound at the intersection of sirtuin and AMPK signaling, has been widely studied in fibrotic and cardiovascular disease and increasingly in cancer. In myocardial infarction and pressure-overload models, resveratrol activates SIRT1/SIRT3, enhances AMPK signaling, and attenuates cardiac fibrosis by suppressing TGF-β/SMAD3 activity and SMAD3 acetylation, thereby reducing collagen I/III expression (Guo et al., 2022[65]; Yarahmadi et al., 2025[191]). In cancer models, resveratrol interferes with TGF-β-driven EMT and invasion, in conjunction with ERK, NF-κB and ROS pathways (Ashrafizadeh et al., 2020[2]). These pleiotropic actions may enable resveratrol or optimized derivatives to target both tumor and stromal compartments in TGF-β-mediated malignancies, although their indirect mechanisms and modest potency raise translational concerns regarding dosing and specificity

Therapeutically, metformin and berberine have the strongest preclinical support for suppressing TGF-β-driven cancer phenotypes through AMPK-related mechanisms. Next steps should focus on robust in vivo tumor models that include relevant stromal elements, biomarker-based stratification of patients by TGF-β and AMPK pathway activity, and rational combinations with immune checkpoint inhibitors or direct TGF-β receptor inhibitors. These integrative studies are needed to move AMPK activators from experimental TGF-β modulators toward clinically useful components of cancer therapy.

#### PPAR agonists as modulators of TGF-β signaling in cancer

Peroxisome proliferator-activated receptors (PPARs), nuclear receptors comprising the α, γ, and β/δ isoforms, integrate lipid and energy metabolism with inflammatory and fibrotic signaling, and their crosstalk with TGF-β has emerged as a key node at the interface of metabolism, fibrosis, and malignancy. Among these, PPARγ is best characterized: classical ligands including the endogenous prostanoid 15-deoxy-Δ¹²,¹⁴-prostaglandin J₂ (15d-PGJ₂), thiazolidinediones such as rosiglitazone and pioglitazone, and synthetic triterpenoids such as CDDO consistently suppress TGF-β-induced fibroblast-to-myofibroblast differentiation in primary human lung fibroblasts, reducing α-smooth muscle actin and collagen expression and inhibiting stress fiber formation (Table 5(Tab. 5); References in Table 5: Bansal et al., 2017[5]; Calvier et al., 2017[18]; Chen et al., 2012[29]; Derrett-Smith et al., 2021[46]; Kikuchi et al., 2021[90]; Kulkarni et al., 2011[93]; Legchenko et al., 2018[102]; Li et al., 2006[105]; Luo et al., 2014[115]; Paw et al., 2023[137]; Zeng et al., 2025[194]). Mechanistically, PPARγ activation largely acts through SMAD-independent inhibition of TGF-β-driven PI3K/Akt and focal adhesion kinase (FAK) signaling, thereby restraining acquisition of a contractile, profibrotic phenotype-pathways that are equally operative in cancer-associated fibroblasts, making PPARγ agonists attractive candidates for repurposing as anti-TGF-β agents in TGF-β-high tumors (Kulkarni et al., 2011[93]).

Rosiglitazone provides a concrete example of how a TZD-type PPARγ agonist can intersect canonical and non-canonical TGF-β signaling. In human Tenon's capsule fibroblasts, a clinically important model of ocular scarring, TGF-β drives transdifferentiation to myofibroblasts via p38 MAPK; rosiglitazone attenuates p38 phosphorylation, reduces α-SMA, CTGF and collagen expression, and dampens cell migration and contractility (Luo et al., 2014[115]). Similar antifibrotic effects have been reported in corneal fibroblasts, where 15d-PGJ₂, troglitazone and rosiglitazone consistently suppress TGF-β-induced α-SMA, collagen I and fibronectin by interfering with TGF-β/p38 signaling. Although these studies were performed in non-malignant tissues, the same TGF-β-p38 axis contributes to EMT and invasion in many carcinomas; thus, TZD-type PPARγ agonists may simultaneously modulate stromal scarring and tumor cell plasticity in TGF-β-high cancers such as hepatocellular carcinoma, pancreatic ductal adenocarcinoma and certain lung cancers (Jeon et al., 2014[83]).

Beyond classical ligands, newer PPARγ-activating scaffolds further clarify how PPARγ directly suppresses the TGF-β/SMAD axis. Lathyrol, a diterpenoid PPARγ agonist, activates and drives nuclear accumulation of PPARγ, promotes PPARγ-Nedd4-dependent ubiquitination and degradation of phosphorylated SMAD3, reduces nuclear SMAD3, and consequently attenuates TGF-β/SMAD signaling and fibroblast-to-myofibroblast transition in vitro as well as bleomycin-induced lung fibrosis in vivo (Zeng et al., 2025[194]). Synthetic triterpenoids such as CDDO, which act as high-affinity PPARγ ligands but also engage Nrf2 and other stress pathways, likewise inhibit TGF-β-induced myofibroblast differentiation and collagen synthesis in human lung fibroblasts, largely by blocking PI3K/Akt and downstream profibrotic nodes required for sustained TGF-β/SMAD activity (Kulkarni et al., 2011[93]). Together, these PPARγ-biased agents provide mechanistic templates for molecules that more directly silence SMAD-dependent transcription and TGF-β-driven EMT while concomitantly modulating cellular stress responses.

PPARα and PPARδ agonists offer complementary means to restrain TGF-β-driven stroma. Fenofibrate inhibits TGF-β-induced myofibroblast differentiation in IMR-90 lung fibroblasts, reducing α-SMA, CTGF, collagen production, SMAD3 phosphorylation and nuclear translocation, and TGF-β-driven metabolic reprogramming, resulting in a globally less profibrotic phenotype, whereas the triterpenoid PPARα agonist arjunolic acid more clearly via PPARα inhibits non-canonical TGF-β signaling (TAK1/p38, NF-κB) and regresses established cardiac fibrosis, a mechanism directly relevant to TGF-β-driven inflammation and EMT in cancer. In primary bronchial fibroblasts from asthmatic patients, the PPARδ agonist GW501516 suppresses TGF-β-induced fibroblast-to-myofibroblast transition, decreasing α-SMA, collagen I/III and fibronectin, reducing SMAD3 phosphorylation and nuclear entry (Paw et al., 2023[137]).

Lanifibranor (IVA337), an oral pan-PPAR (α/δ/γ) agonist, restores PPAR signaling in a fibroblast-specific TβRII-driven systemic sclerosis model, reduces persistent bleomycin-induced lung fibrosis, and improves cardiorespiratory features (Derrett-Smith et al., 2021[46]). Telmisartan, an AT1 receptor blocker with partial PPARγ activity, shows a similar PPAR-linked effect on TGF-β signaling: it reverses TGF-β1-induced EMT in HK-2 cells (restores E-cadherin, lowers α-SMA and CTGF) and decreases TGF-β/SMAD activation and ECM accumulation in diabetic and hypertensive nephropathy models, supporting repurposing or combination use in cancer patients already taking renin-angiotensin system inhibitors.

#### Epigenetic and non-coding nucleic acid-based modulation of TGF-β signaling

Antisense oligonucleotides (ASOs) are short, chemically modified single-stranded nucleic acids that bind target RNA and modulate gene expression through RNase H-mediated degradation or steric blocking. In the TGF-β pathway, ASOs offer isoform-selective mRNA targeting and enable selective inhibition of specific ligands or receptors, making them attractive for cancer therapy where TGF-β signaling contributes to tumor progression and immune evasion (Jing et al., 2025[86]). However, ASOs also have limitations, including delivery challenges, potential off-target effects, limited tissue penetration, and the need for repeated dosing due to transient activity.

Trabedersen (AP 12009, OT-101) is an 18-mer phosphorothioate antisense oligodeoxynucleotide complementary to human TGF-β_2_ mRNA (Schlingensiepen et al., 2008[152]; Jaschinski et al., 2011[82]); by binding the 5′-UTR and triggering RNase H-dependent degradation, it selectively depletes TGF-β_2_ while largely sparing TGF-β_1_ and TGF-β_3_ (Schlingensiepen et al., 2006[154]). Preclinically, trabedersen downregulates TGF-β_2_ in high-grade glioma and pancreatic carcinoma cells, reduces invasive growth, angiogenesis and experimental metastasis, and reverses TGF-β-mediated immunosuppression by decreasing regulatory T cells and myeloid-derived suppressor cells while enhancing cytotoxic T-cell activity, leading to delayed tumor growth and improved survival in rodent glioma and pancreatic cancer models (Schlingensiepen et al., 2011[153]; D'Cruz et al., 2018[43]). Clinically, local (intratumoral or intraventricular) administration in recurrent high-grade glioma achieved sustained TGF-β_2_ suppression and encouraging survival in phase I/II studies (Hau et al., 2007[70]; Bogdahn et al., 2011[11]), supporting a randomized phase IIb trial in recurrent anaplastic astrocytoma and a subsequent phase III program, while systemic intravenous trabedersen in a phase I/II dose-escalation study in advanced pancreatic carcinoma, metastatic melanoma and colorectal carcinoma showed manageable toxicity, disease stabilization in a subset of patients and signals of prolonged survival in pancreatic cancer (Jaschinski et al., 2011[82]). More recently, OT-101 has been repositioned as an immuno-oncologic adjuvant, with a phase I/II study reporting favorable overall survival in pancreatic cancer patients treated with OT-101 followed by chemotherapy, and ongoing trials evaluating combinations with mFOLFIRINOX in pancreatic adenocarcinoma and with PD-1 blockade (e.g., pembrolizumab) in selected solid tumors, collectively supporting selective TGF-β_2_ knockdown as a means to mitigate TGF-β-driven immune evasion and stromal remodeling in TGF-β_2_-overexpressing cancers (D'Cruz et al., 2018[43]; Omar et al., 2025[130]).

ISTH0036, a 14-mer phosphorothioate LNA-gapmer selectively targeting TGF-β_2_ mRNA. In a first-in-human phase I trial in advanced primary open-angle glaucoma undergoing trabeculectomy, a single intravitreal dose was well tolerated and associated with sustained intraocular TGF-β_2_ suppression, prolonged bleb survival and reduced postoperative scarring, with ongoing phase II studies in retinal fibrosis further supporting anti-fibrotic activity (Pfeiffer et al., 2017[141]). Although these indications are non-oncologic, they provide proof-of-principle that potent, selective TGF-β_2_ ASO therapy can be delivered safely and achieve tissue-level anti-fibrotic effects relevant to TGF-β-driven tumor biology (Jing et al., 2025[86]).

In addition to targeting TGF-β ligands, antisense oligonucleotides (ASOs) have been developed against TGF-β receptors and signaling adaptors. ASOs targeting TβRI/ALK5 can reduce receptor expression, dampen SMAD signaling, and lessen fibrotic remodeling in preclinical models, offering another way to suppress the pathway and potentially complement ligand-directed approaches in tumors with strong stromal or endothelial TGF-β activity (Kemaladewi et al., 2014[89]). Applying this strategy in oncology still faces major hurdles, including efficient delivery into tumors, compensatory signaling among TGF-β isoforms, and how best to combine ASOs with chemotherapy, radiotherapy, immune checkpoint blockade, or small-molecule TGF-β receptor inhibitors. Even so, the target specificity of ASOs and their growing clinical track record support continued development as part of combination regimens to selectively blunt TGF-β signaling in cancer.

## Challenges, Opportunities, and Future Outlook

Clinical development of TGF-β inhibitors has been limited by on-target toxicity and narrow therapeutic windows in both extracellular and intracellular approaches. Current strategies emphasize intermittent dosing to reduce cumulative toxicity, combination therapy to lower dose requirements and limit compensatory resistance, and biomarker-based patient selection rather than treating all tumors as equally TGF-β-dependent. In the phase II galunisertib plus sorafenib study in advanced hepatocellular carcinoma, galunisertib was given on a 14-days-on/14-days-off schedule, the combination showed acceptable safety, and on-treatment biomarker changes (including circulating TGF-β_1_) were associated with clinical outcome (Kelley et al., 2019[88]). Together, these results support schedule- and biomarker-informed use of TGF-β inhibitors instead of continuous, broad suppression in unselected patients.

An important opportunity is to move further downstream or into regulatory layers that can suppress pro-tumor signaling without fully blocking all TGF-β functions in all tissues. One example is nucleocytoplasmic transport: Smad signaling depends on regulated nuclear import and export, and studies on Smad nucleocytoplasmic shuttling and transport control support pharmacologic interference with this process to alter TGF-β/Smad signaling output (Kurisaki et al., 2006[94]). This approach is attractive because nuclear transport inhibitors may also affect nuclear trafficking of multiple oncogenic factors beyond SMADs. Another opportunity is to target intracellular trafficking and receptor routing. Compounds that shift receptor trafficking toward sequestration or degradation may suppress not only TGF-β receptors but also additional pro-tumor receptors that share trafficking machinery, creating a multi-pathway inhibitory effect through one trafficking-centered mechanism; the sorafenib study showing membrane-domain-dependent TβRII depletion and cell-dependent effects provides a mechanistic precedent for this concept.

Drug repurposing is a practical way to expand strategies for modulating TGF-β-related cancer phenotypes, and fluoroquinolones provide a representative example of concentration- and mechanism-dependent effects. At lower concentrations, fluoroquinolones can induce IGFBP-3 and inhibit IGF signaling (Chung and Chen, 2024[38]), and can also increase betaglycan expression through EPAC (RAPGEF3), thereby suppressing migration and metastasis (Liu et al., 2025[110]); At higher concentrations, fluoroquinolones can inhibit DNA synthesis, suppress proliferation, and induce apoptosis in carcinoma cells (Herold et al., 2002[73]). These effects are mechanistically relevant because increased betaglycan can inhibit invasion through TGF-β-dependent ligand sequestration (including soluble betaglycan) and TGF-β-independent mechanisms, whereas IGFBP-3 signaling through LRP1 (TβR-V)-identified in several studies as a receptor required for IGFBP-3/TGF-β growth-inhibitory signaling in epithelial cells-provides an additional growth-suppressive pathway (Huang et al., 2003[79]). In addition, noncanonical endogenous regulators may provide new therapeutic entry points, as tyrosine hydroxylase has been shown to interact with SMAD2 and suppress TGFβ/Smad signaling in hepatocellular carcinoma (Liu et al., 2024[109]). Likewise, some kinase inhibitors developed for other targets can also suppress TGF-β pathway activity; for example, PLX8394 inhibits TGF-β signaling, invasion, and tumor growth in cutaneous squamous cell carcinoma models, supporting multi-target strategies when concurrent inhibition of MAPK- and TGF-β-associated signaling is biologically justified (Siljamaki et al., 2023[163]). Together, these findings support a future direction in which TGF-β pathway inhibition is implemented through mechanism-guided combinations, biomarker-based selection, and repurposed or multi-target agents that suppress pro-tumor signaling while limiting toxicity from global pathway blockade.

## Conclusion and Future Perspective

TGF-β-targeted therapy in cancer will not be advanced by broader pathway blockade alone. Progress will require context-matched intervention at different regulatory levels, including ligand/receptor inhibition, receptor trafficking and compartmentalization, nucleocytoplasmic transport, post-translational regulation, and downstream signaling control. This approach also supports drug repurposing and multi-target strategies that suppress pro-tumor TGF-β signaling while limiting toxicity from global pathway inhibition. Future work should focus on mechanism-guided combinations, biomarker-based patient selection, and context-specific pharmacodynamic readouts to achieve more consistent clinical benefit.

## Notes

Shun-Ban Tai and Jenq-Lin Yang contributed equally as first author.

## Declaration

### Acknowledgment

This study was supported by the National Science and Technology Council of Taiwan (NSTC 114-2320-B-110 -003 -MY3, 113-2320-B-110-006, 111-2320-B-110-008), the NSYSU-KMU Joint Research Project (NSYSUKMU-115-P06), Zuoying Armed Forces General Hospital (KAFGH-ZY_A_113002, KAFGH-ZY_A_110002). Kaohsiung Armed Forces General Hospital Joint Research Program (KAFGH_A_113006), CMMC-NSYSU Joint Research Program 113-04, Innovation Center for Drug Development and Optimization, National Sun Yat-sen University, and the Chang Gung Medical Foundation CMRPG8P0121.

### Conflict of interest 

The authors declare no conflict of interest in this study.

### Artificial Intelligence (AI) - assisted technology

Artificial intelligence tools were used only for minor language editing, including grammar correction and typo checking. No AI tools were used to generate scientific content, interpret data, or draw conclusions.

### Author contribution

Shun-Ban Tai and Jenq-Lin Yang collected and curated the literature and prepared the tables. Pei-Feng Liu checked the manuscript for errors and prepared the figures. Chun-Lin Chen wrote and revised the manuscript and served as the corresponding author. All authors reviewed and approved the final manuscript. All data were generated internally, and no external writing assistance or paper mill services were used. All authors agree to be accountable for all aspects of the work to ensure its integrity and accuracy.

## Figures and Tables

**Table 1:**
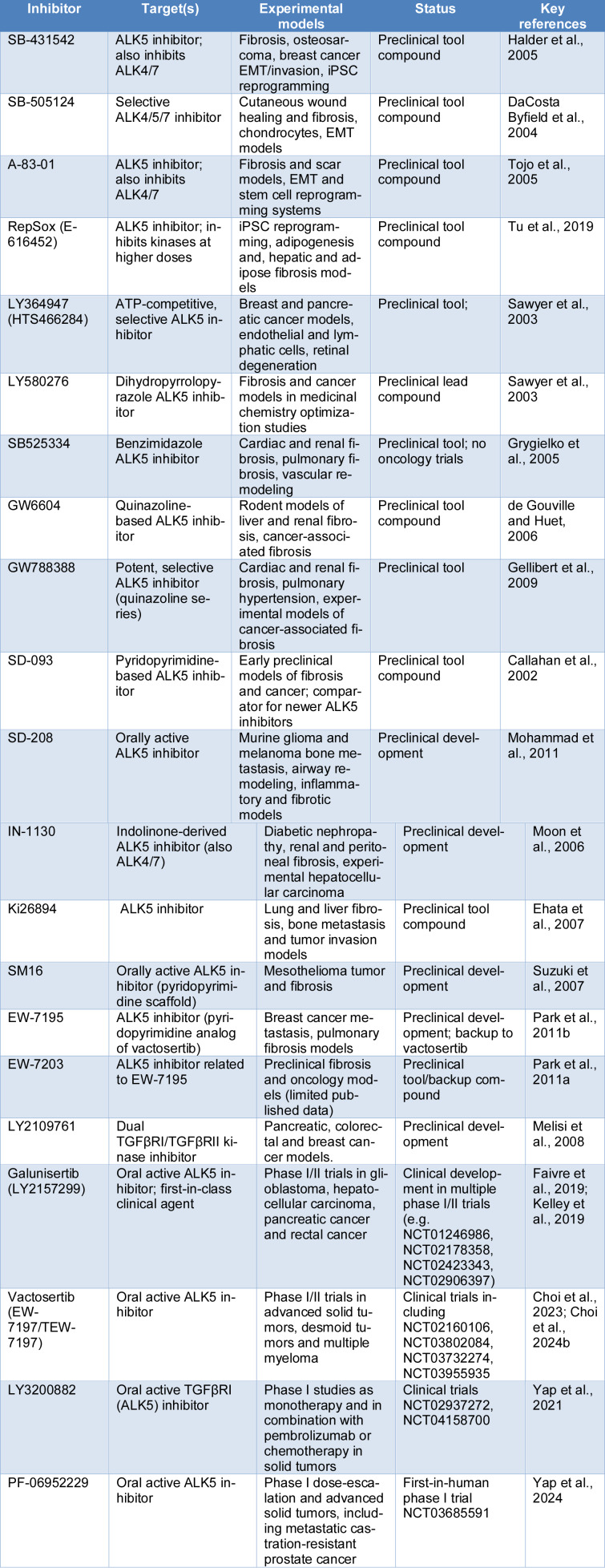
TGF-β receptor kinase inhibitors (including tool compounds)

**Table 2:**
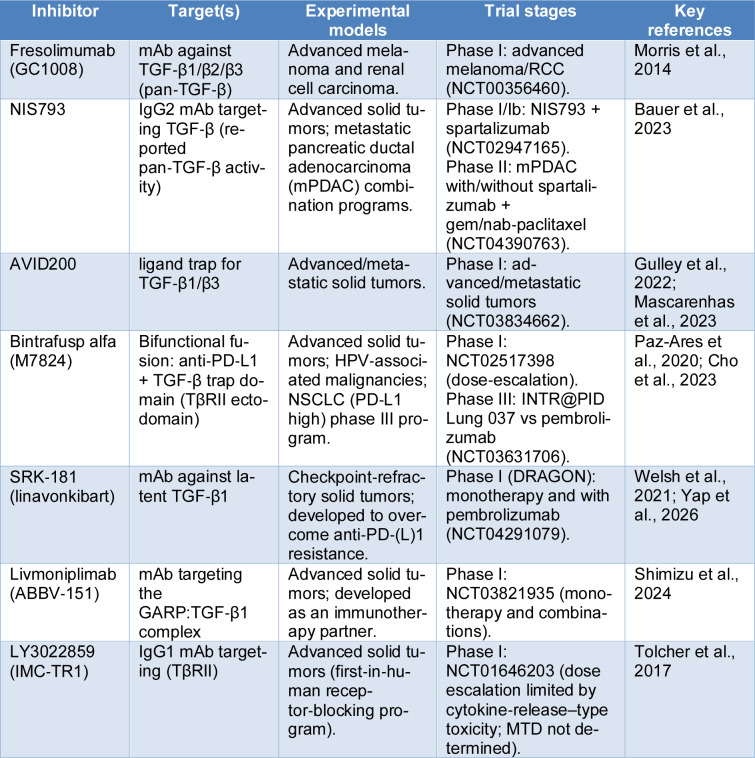
TGF-β ligand-trap and receptor-binding blockade

**Table 3:**
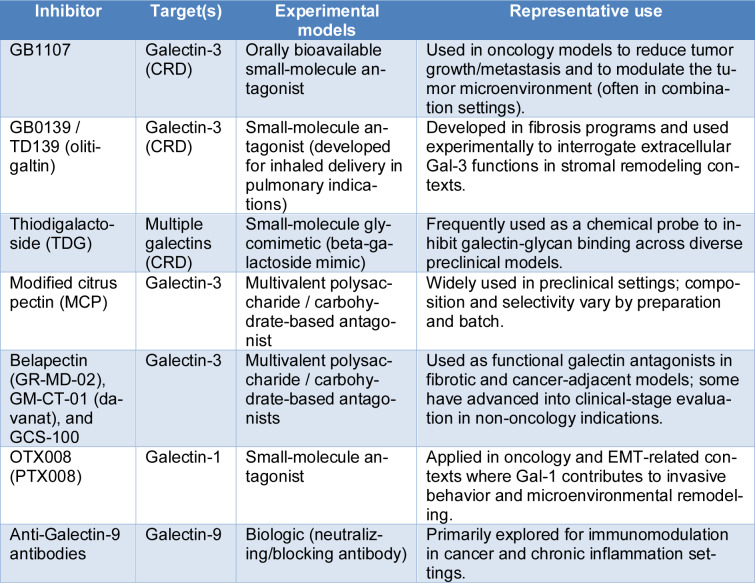
Galectin inhibitors intersecting the TGF-beta axis

**Table 4:**
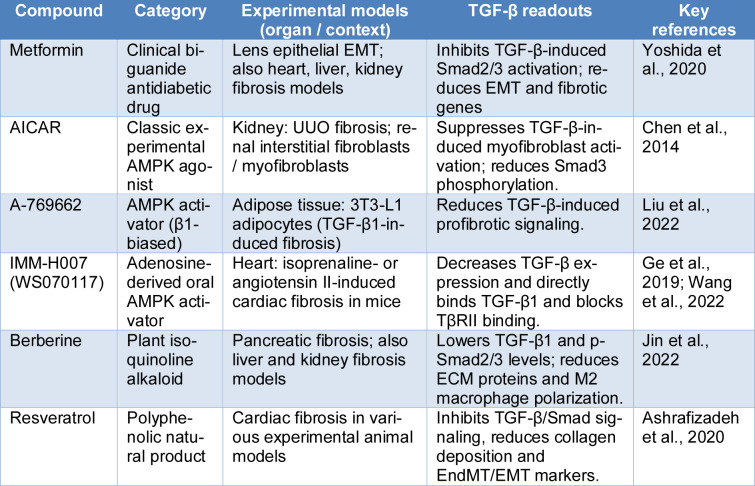
AMPK Activators that suppress TGF-β/Smad signaling

**Table 5:**
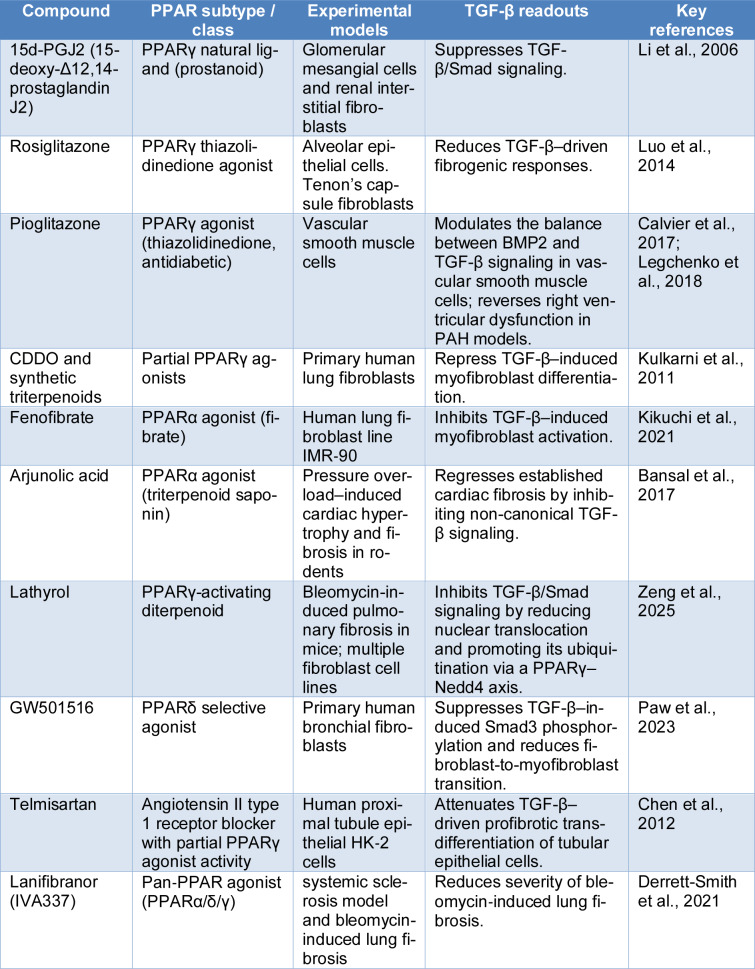
PPAR agonists that Inhibit TGF-β signaling
